# Acquisition and decay of IgM and IgG responses to merozoite antigens after *Plasmodium falciparum* malaria in Ghanaian children

**DOI:** 10.1371/journal.pone.0243943

**Published:** 2020-12-17

**Authors:** Melanie R. Walker, Anne S. Knudsen, Frederica D. Partey, Maria R. Bassi, Asger M. Frank, Filip C. Castberg, Edem W. Sarbah, Michael F. Ofori, Lars Hviid, Lea Barfod

**Affiliations:** 1 Department of Immunology and Microbiology, Centre for Medical Parasitology, Faculty of Health and Medical Sciences, University of Copenhagen, Copenhagen, Denmark; 2 Noguchi Memorial Institute for Medical Research, University of Ghana, Legon, Ghana; 3 Department of Clinical Microbiology, Copenhagen University Hospital (Rigshospitalet), Copenhagen, Denmark; Universidade Federal de Minas Gerais, BRAZIL

## Abstract

Developing a vaccine against *Plasmodium falciparum* malaria has been challenging, primarily due to high levels of antigen polymorphism and a complex parasite lifecycle. Immunization with the *P*. *falciparum* merozoite antigens PfMSRP5, PfSERA9, PfRAMA, PfCyRPA and PfRH5 has been shown to give rise to growth inhibitory and synergistic antisera. Therefore, these five merozoite proteins are considered to be promising candidates for a second-generation multivalent malaria vaccine. Nevertheless, little is known about IgG and IgM responses to these antigens in populations that are naturally exposed to *P*. *falciparum*. In this study, serum samples from clinically immune adults and malaria exposed children from Ghana were studied to compare levels of IgG and IgM specific for PfMSRP5, PfSERA9, PfRAMA, PfCyRPA and PfRH5. All five antigens were found to be specifically recognized by both IgM and IgG in serum from clinically immune adults and from children with malaria. Longitudinal analysis of the latter group showed an early, transient IgM response that was followed by IgG, which peaked 14 days after the initial diagnosis. IgG levels and parasitemia did not correlate, whereas parasitemia was weakly positively correlated with IgM levels. These findings show that IgG and IgM specific for merozoite antigens PfMSRP5, PfSERA9, PfRAMA, PfCyRPA and PfRH5 are high in children during *P*. *falciparum* malaria, but that the IgM induction and decline occurs earlier in infection than that of IgG.

## Introduction

*Plasmodium falciparum* malaria is estimated to cause more than 400,000 deaths annually [1]. In sub-Saharan Africa, where *P*. *falciparum* is rampant, young children are particularly vulnerable to the disease. This is primarily because they lack protective immunity [2] which is gradually acquired with age as a result of repeated exposure to the parasite. This acquisition eventually leads to control of blood-stage parasitemia and clinical symptoms [3–6].

Parasite resistance to antimalarial drugs and insecticides poses a major threat [7–9] and many challenges remain to achieve global elimination of malaria, including the need to develop a prophylactic malaria vaccine. Developing a vaccine against *P*. *falciparum* malaria has proved difficult primarily due to high levels of antigen polymorphism and a complex parasite lifecycle [10–12]. To date, only the RTS,S vaccine has reached phase III trials, where it was found to have only 26% to 36% efficacy [13]. Nevertheless, RTS,S has recently been advanced to large-scale post-licensing pilot trials in Africa [14]. However, its modest efficacy and lack of strain-transcendent immunity [15, 16] means that the development of a second-generation malaria vaccine remains a major priority. As naturally acquired immunity (NAI) to malaria is primarily directed against blood stage parasite antigens, merozoite proteins are thought to be suitable candidates for a second-generation vaccine [17, 18].

Immunoglobulin G (IgG) is thought to play a major role in NAI [19, 20], however limited studies have investigated the role of *P*. *falciparum*-specific IgM in protective immunity [21–24]. The most informative study to date suggests that merozoite-specific IgM is rapidly induced in malaria-naïve adults, is prominent in *P*. *falciparum*-infected children and adults, and is long-lived in the absence of re-infection [25]. Furthermore, *Plasmodium-*specific IgM memory B cells have been found in naturally exposed human subjects and in mouse models of malaria infection [26].

Understanding IgM and IgG acquisition and maintenance is important for the development of antibody-based vaccines. IgM has been shown to be critical for protection against multiple human pathogens including West Nile virus, HIV, Hepatitis C virus, chikungunya and influenza viruses [27–31]. Furthermore, an early neutralizing IgM response is induced following immunization with the smallpox (vaccinia virus) vaccine, one of the most successful vaccines to date [32].

The *P*. *falciparum* merozoite antigens PfMSRP5, PfSERA9, PfRAMA, PfCyRPA and PfRH5 have recently been identified as promising vaccine candidates [33]. This is due to epidemiological data showing that combinations of antibodies against these targets are associated with protection [33, 34]. These antigens are also able to induce growth inhibiting antibodies that have synergistic interactions *in vitro* when combined [33]. Furthermore, a vaccine candidate based on PfRH5 – an antigen that plays a crucial role in merozoite invasion of erythrocytes by binding to the host receptor basigin and initiating parasite entry [35–38] - is currently in phase 1/IIa clinical trials [39–41]. Nevertheless, no study to date has investigated both IgG and IgM responses specific for these five merozoite antigens in both adults and children naturally exposed to malaria [33, 41–47].

Previously, we and others have shown that IgG responses are induced against PfRH5 and PfCyRPA during *P*. *falciparum* malaria [41, 47]. In the current study, we expand on these findings and examine IgM responses towards five merozoite antigens (PfMSRP5, PfSERA9, PfRAMA, PfCyRPA PfRH5), and IgG responses towards three merozoite antigens (PfMSRP5, PfSERA9 and PfRAMA) in naturally exposed Ghanaian adults and children with distinct clinical characteristics. Our findings indicate that both IgM and IgG are present in clinically immune adults and in children with acute *P*. *falciparum* malaria. Furthermore, we show, that in children IgM induction and decline towards PfMSRP5, PfSERA9, PfRAMA, PfCyRPA and PfRH5 occurs earlier in infection than IgG.

## Materials and methods

### Subjects and samples

Samples from adults were collected from Asutsuare, a town within the Shai-Osudoku district, Greater Accra Region, from April – June 2014, as previously described [41]. Samples from children (aged 1-12) were collected from Hohoe, Ghana, an area of high *P*. *falciparum* malaria transmission intensity, from May – August 2015, as described previously [47, 48].

The cohorts collected here are representative of the area in which they were collected. Study participants were enrolled on admission at Hohoe municipal hospital (Febrile children) or from within the Hohoe and Asutsauare communities after engagement with community/opinion leaders. This was followed by a durbar in the community to explain the study in the local language to the residents [48]. Subjects were recruited after informed written consent had been obtained; in the case of children, from a parent or legal guardian. Children and adults were excluded from the study if the individual had a major medical condition or were sickling positive. Pregnant women were also excluded from the study. All sample recruitment, collection and preparation was performed in Ghana. The screening of samples for merozoite antigen-specific antibodies was performed at the University of Copenhagen, Copenhagen, Denmark.

In brief, 78 healthy adult (18 – 69 years) male and female volunteers who were likely to have been exposed on numerous occasions to *P*. *falciparum* but were malaria negative by rapid diagnostic test (RDT) and microscopy were recruited from within the Asutsuare community and included in the study **(Table 1)** [41]. Febrile children (n = 105) with an oral temperature > 37.5°C and aged 1-12 were recruited from Hohoe municipal hospital. Malaria patients (n = 95) were defined as those where *P*. *falciparum* malaria was confirmed by a positive rapid RDT and by light microscopy with a parasite density > 2,500 parasites/μL. These malaria patients were divided into those with severe malaria (SM, n = 39) or uncomplicated malaria (UM, n = 56), respectively, as previously described [47]. Febrile patients without detectable parasitemia were grouped as febrile controls (FC, n = 10) **(Table 1)**. All children were treated according to Ghanaian medical treatment guidelines. Children with SM and UM were seen again at 14 days (Day 14) and 42 days (Day 42) after the initial day of diagnosis (Day 0). In addition, 85 clinically healthy, age-matched children from within the Hohoe community were recruited in a similar manner, and grouped as either asymptomatic (AC, n = 29) (where RDT positivity was observed with the addition of some subjects having low parasitemia (<2,000/μL)) or uninfected (HC, n = 56) controls (where children were negative by microscopy and RDT) **(Table 1)**.

**Table 1:** Demographic characteristics of subjects.

Category	SM^a^	UM	FC	AC	HC	CI
****sex ratio (F/M)****	10/29	27/29	1/9	15/14	29/27	38/40
****Age (years)****	5 (1-8)^b^	4 (1-12)	5 (2-12)	6 (1-10)	5 (1-10)	33 (18-69)
****Haemaglobin****	7.8 (6.2–11.8)	10 (6.3–13.8)	11.1 (6.3–13)	11 (6.5–12.8)	11.6 (4.2–11.6)	12.8 (8.3–18.2)
****Parasitaemia****	52200 (2600–1600800)	38200 (2900–649000)	0	1370 (300–1900)	0	0

^a^SM (severe *P*. *falciparum* malaria), UM (uncomplicated *P*.*falciparum* malaria), FC (febrile controls), AC (asymptomatic controls), HC (healthy controls), CI (Clinically immune adults).

^b^Median (range).

### Ethics statement

Human research ethics approvals were obtained from the Ethics Committee of the Noguchi Memorial Institute for Medical Research, University of Ghana (NMIMR-IRB: CPN: 010/12-13) and from the Ghana Health Service (GHS-ERC 08/05/14). Plasma samples for the 10 non-exposed, anonymous Danish adults used as negative controls in the ELISAs were approved by the Regional Research Ethics Committees for the Capital Region of Denmark (Protocol H-4-2013-083). All methods were performed in accordance with the relevant guidelines and regulations.

### *P*. *falciparum* merozoite antigen proteins

Recombinant PfRH5, sourced from Prof. Simon J Draper (University of Oxford, Oxford, UK), was expressed using a *Drosophila melanogaster* Schneider 2 stable cell line system as previously described [49].

Recombinant extracellular domains of merozoite antigens PfMSRP5-bio-his (Addgene plasmid #50805), PfSERA9-bio-his (Addgene plasmid #50820), PfRAMA-bio-his (Addgene plasmid #50737), and PfCyRPA-bio-his (PFD1130w-bio-his, Addgene plasmid #50823) were gifts from Dr. Gavin Wright (University of Oxford, Oxford, UK) [45]. Transient transfection of expression plasmids was performed using the Expi293F^TM^ Expression System Kit (ThermoFisher) as per manufacturer’s instructions. Media was harvested four days post transfection for purification. This supernatant was then passed through a 5 mL HisTrap HP chromatography column (GE Healthcare) charged with 0.1M NiSO_4_.6H_2_O and washed with sodium phosphate buffer with 25 mM Imidazole. Proteins were eluted from the column by passing through 500 mM imidazole in sodium phosphate buffer. Elution fractions were pooled and buffer exchanged into PBS. Quality of antigens were assessed using SDS-PAGE and western blotting performed with Anti-his-HRP (C-term) antibody (Miltenyi Biotec).

### Enzyme linked immunoassays

Microtiter plates, 96 well (Nunc Maxisorb, ThermoFisher Scientific) were prepared with 2 μg/mL of either PfMSRP5, PfSERA9, PfRAMA, PfCyRPA or PfRH5 and incubated overnight. Plates were washed three times with PBS-T and then blocked for one hour with blocking buffer (5% non-fat dry milk in PBS-T). The bound antigen was then incubated with plasma at a final dilution of 1:100 for 1 hour, followed by a 1 hour incubation with anti-human IgG-AP (Sigma Aldrich, 1:1500) or anti-human IgM-AP (Sigma Aldrich, 1:1500). Next, an incubation with 4-Nitrophenyl phosphate disodium salt hexahydrate tablets (Sigma Aldrich) dissolved in 1 × Diethanolamine Substrate Buffer (Sigma Aldrich) was performed. Color development and absorbance were measured at 405 nm.

Plasma pools of semi-immune adults against each antigen were used as positive controls and to normalize plate to plate variability. Antibody levels were presented as arbitrary units (AU) calculated as (OD_sample_-OD_blank_)/ (OD_positive control_ -OD_blank_). In order to define a true positive result, a cut-off value was calculated for each assay as the mean + 3SD of OD values in plasma from 10 healthy Danish donors.

To measure breadth and magnitude of the individual plasma samples, a breadth score was calculated. Positive antibody responses to each antigen were stratified into tertiles and binding against a specific antigen was given a score of 1, 2 or 3 for the lowest, second and highest tertile, respectively [50]. Samples that were negative were given a score of 0. The breadth score was calculated as the sum of scores for all antigens assessed for that plasma sample.

### Statistical analysis

Data analysis was performed and graphs created using GraphPad Prism Software (version 7.0, GraphPad). A Kruskal-Wallace and Dunn’s post-hoc test were used to evaluate antibody levels in the different donor groups, and a Friedman and Dunn’s post-hoc test for longitudinal samples. Fisher’s exact tests were performed to compare proportions. A Wilcoxon matched-pairs signed rank test was performed to compare IgM and IgG levels. Spearman’s correlation co-efficient were performed to compare; IgM and IgG levels to merozoite antigens, antibody levels to merozoite antigens, antibody levels to age, and antibody levels and log-transformed parasitemia. Principle component analysis was performed with the ggbiplot package in R (v3.3.1; The R Foundation for Statistical Computing) using default parameters. For each IgG and IgM reactivity combination, the parasitemia (log10) were compared for positive and negative responders using Wilcoxon test and Benjamini–Hochberg adjusted P values (false discovery rates). The analysis was limited to antigen combinations for which there were at least six positive and negative responders. Analysis was performed using R and GraphPad Prism Software. Temporal changes in antibody levels were evaluated using cohort running means as previously described [47]. Linear regression analysis was performed on predicted antibody declines as well as observed antibody declines. Slopes of the regression analysis were then compared (Graphpad Prism). Throughout all analysis, statistical significance was defined as a p value less than 0.05 and results expressed as mean ± standard deviation (SD).

## Results

### PfMSRP5, PfSERA9, PfRAMA, PfCyRPA and PfRH5 are immunologically recognized by IgM and IgG in children with *P*. *falciparum* malaria

**Fig 1:**
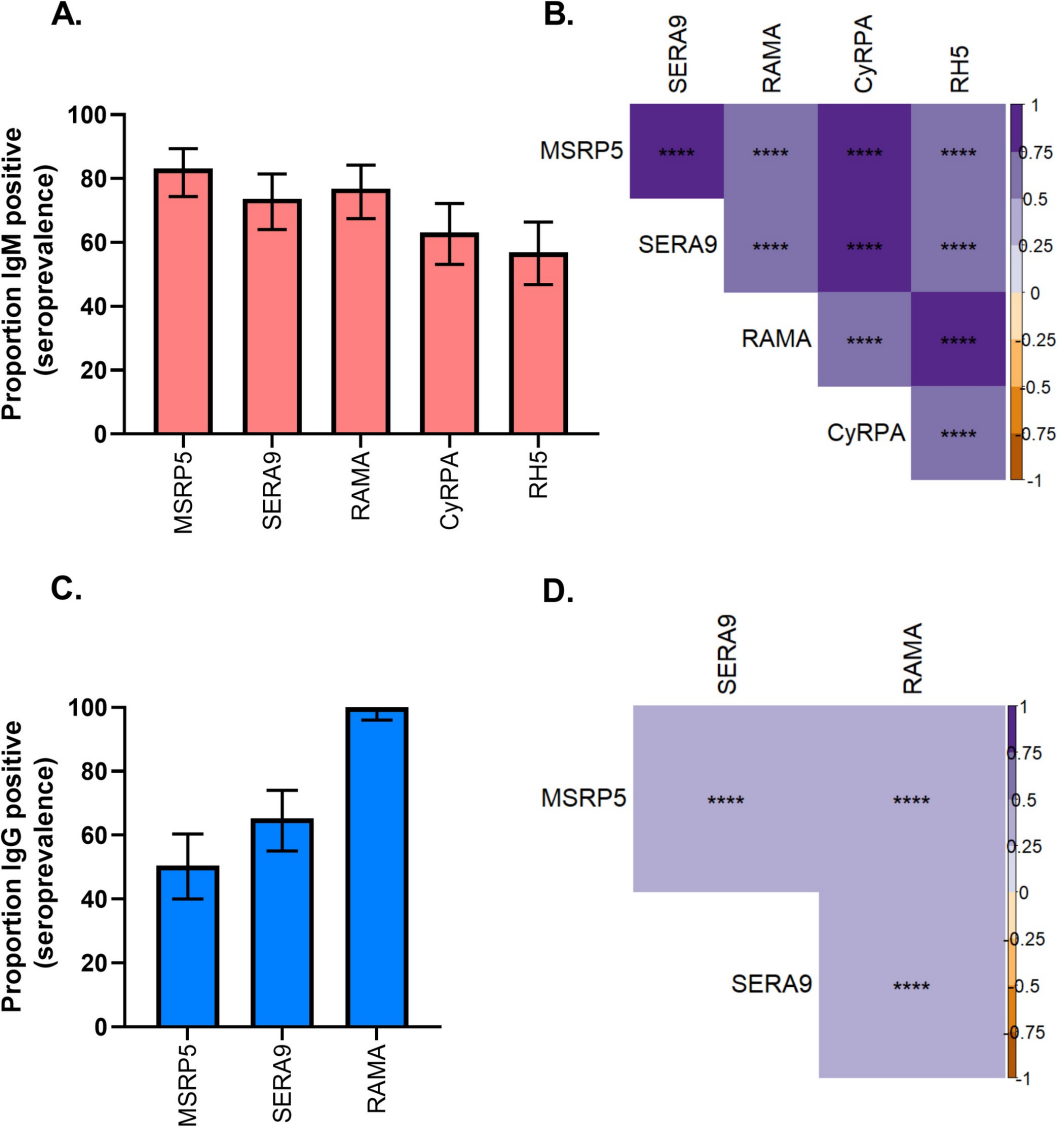
Prevalence and correlation of IgM, and IgG specific for merozoite antigens in children with *P*. *falciparum* malaria. PfMSRP5, PfSERA9, PfRAMA, PfCyRPA and PfRH5 -specific IgM and IgG responses in children with acute *P*. *falciparum* malaria were measured by ELISA. Percent of samples with merozoite antigen-specific IgM (red), and IgG (blue), and their 95% confidence intervals (error bars) are shown in panels A. and C., respectively. Spearman’s correlation of antibody responses between the antigens is shown for IgM (B.) and IgG (D.). Statistical significance is represented by asterisks (p<0.05 (*), p<0.01 (**), p<0.001 (***), p<0.0001 (****)) and color represents the strength and direction of correlation as shown in the key.

Recently, merozoite-specific IgM has been found to be important in NAI against malaria [25]. However, to date no study has investigated IgM responses to PfMSRP5, PfSERA9, PfRAMA, PfCyRPA and PfRH5. We first investigated the prevalence of IgM specific for these five antigens in the plasma of 95 children with confirmed *P*. *falciparum* malaria. At baseline (Day 0), more than two thirds of the children had IgM specific for PfMSRP5 (83.2%), PfSERA9 (73%) and PfRAMA (76.8%) **(Fig 1A)**. The prevalence of IgM specific for PfCyRPA and PfRH5 was slightly lower, with 63.2% having PfCyRPA-specific IgM, and 56% having PfRH5-specific IgM **(Fig 1A)**. IgM responses for all antigen combinations tested were all significantly positively correlated **(Fig 1B)**, suggesting that these antigens are immunologically regulated in a similar way following *P*. *falciparum* malaria.

We next investigated whether IgG is induced against PfMSRP5, PfSERA9, and PfRAMA. PfCyRPA and PfRH5 IgG responses were not examined as these have been previously reported [47]. Half of the subjects (50.5%) had PfMSRP5-specific IgG, two thirds (65.3%) had PfSERA9-specifc IgG, while all (100%) had PfRAMA-specific IgG **(Fig 1C)**. When IgG responses were assessed for correlation between antigens, significant positive correlation was observed for PfMSRP5, PfSERA9 and PfRAMA **(Fig 1D)**.

The analyses above confirm that both IgG and IgM to each of the merozoite antigens tested are induced following natural exposure to malaria. These results support the previously published data on PfMSRP5, PfSERA9 and PfRAMA -specific IgM and IgG [25, 33].

### Merozoite antigen-specific IgM and IgG are higher in children with *P*. *falciparum* malaria than in children without *P*. *falciparum* malaria

To understand the impact of recent parasite exposure, and to determine whether Ig isotypes towards specific merozoite antigens are associated with protection, as has been reported for other merozoite antigens [17, 25, 34, 51, 52], we sub-divided the children with acute *P*. *falciparum* malaria into two groups: uncomplicated (UM) and severe malaria (SM). Furthermore, we tested IgG and IgM responses against the five merozoite antigens in clinically immune adults (CI), and in children from three additional distinct clinical groups: healthy control children (HC), febrile control children (FC), and asymptomatic control children (AC) (see materials and methods). Clinically immune adults were included to determine IgM and IgG responses in subjects with a lifetime of natural malaria exposure.

**Fig 2:**
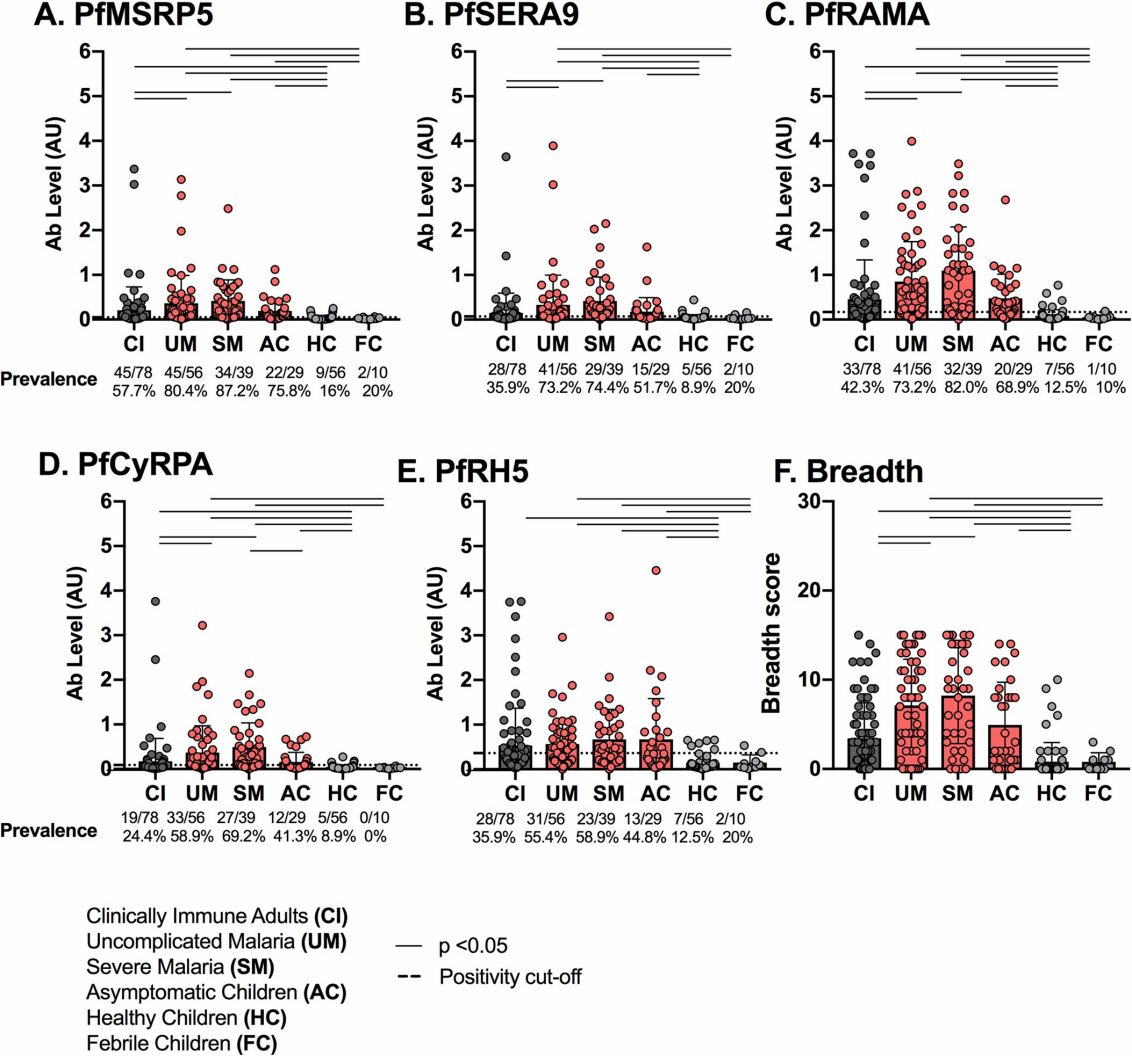
Prevalence and levels of IgM to *P*. *falciparum* merozoite antigens per clinical group. Levels (AU, mean with SD) of IgM specific for PfMSRP5 (A.), PfSERA9 (B.), PfRAMA (C.), PfCyRPA (D.) and PfRH5 (E.) were measured in clinically immune adults (CI, dark grey), children with *P*. *falciparum* malaria (asymptomatic children (AC), uncomplicated malaria (UM), severe malaria (SM), red) and children without *P*. *falciparum* malaria (healthy children (HC), febrile children (FC), light grey). A Kruskal-Wallace test and Dunn’s post-hoc test were performed between all possible combinations of two clinical groups. Combinations with statistical significance only (p < 0.05) are represented by horizontal black lines. All p values regardless of statistical significance are shown in **S1 Table in S1 File.** Positivity cut-offs are represented by a dashed black line. For each clinical group, the number of positive individuals, total number of individuals tested and percent reactivity is shown at the bottom of each panel.

All merozoite antigens tested were recognized by IgM from all the clinical categories **(Fig 2)**. Overall, IgM prevalence and IgM levels in children without *P*. *falciparum* malaria (FC and HC) were significantly lower than those in children with *P*. *falciparum* malaria (AC, SM, UM) **(Fig 2, S1 Table in S1 File)**. Furthermore, for all antigens, IgM prevalence and IgM levels (with the exception of PfRH5 levels) in clinically immune adults were significantly lower than in children with malaria (UM and SM) **(Fig 2A–2D, S1 Table in S1 File)**. Together, these results indicate that levels of IgM specific for merozoite antigens PfMSRP5, PfSERA9, PfRAMA and PfCyRPA are higher in children with *P*. *falciparum* malaria than in those without.

Immunity to malaria is thought to result from high antibody titers (magnitude) to multiple antigens (breadth) [50]. Therefore, a breadth score was calculated to account for both breadth and magnitude. Breadth scores of groups were then compared to determine whether breadth and/or magnitude were important predictors of clinical protection **(Fig 2F, S1 Table in S1 File)**. Breadth analysis of IgM showed that children with acute *P*. *falciparum* malaria (SM and UM) had a significantly broader response than clinically immune adults (CI) and uninfected children (HC and FC). These results further indicate that children with acute *P*. *falciparum* malaria have higher antibodies specific for selected merozoite antigens than children without *P*. *falciparum* malaria.

**Fig 3:**
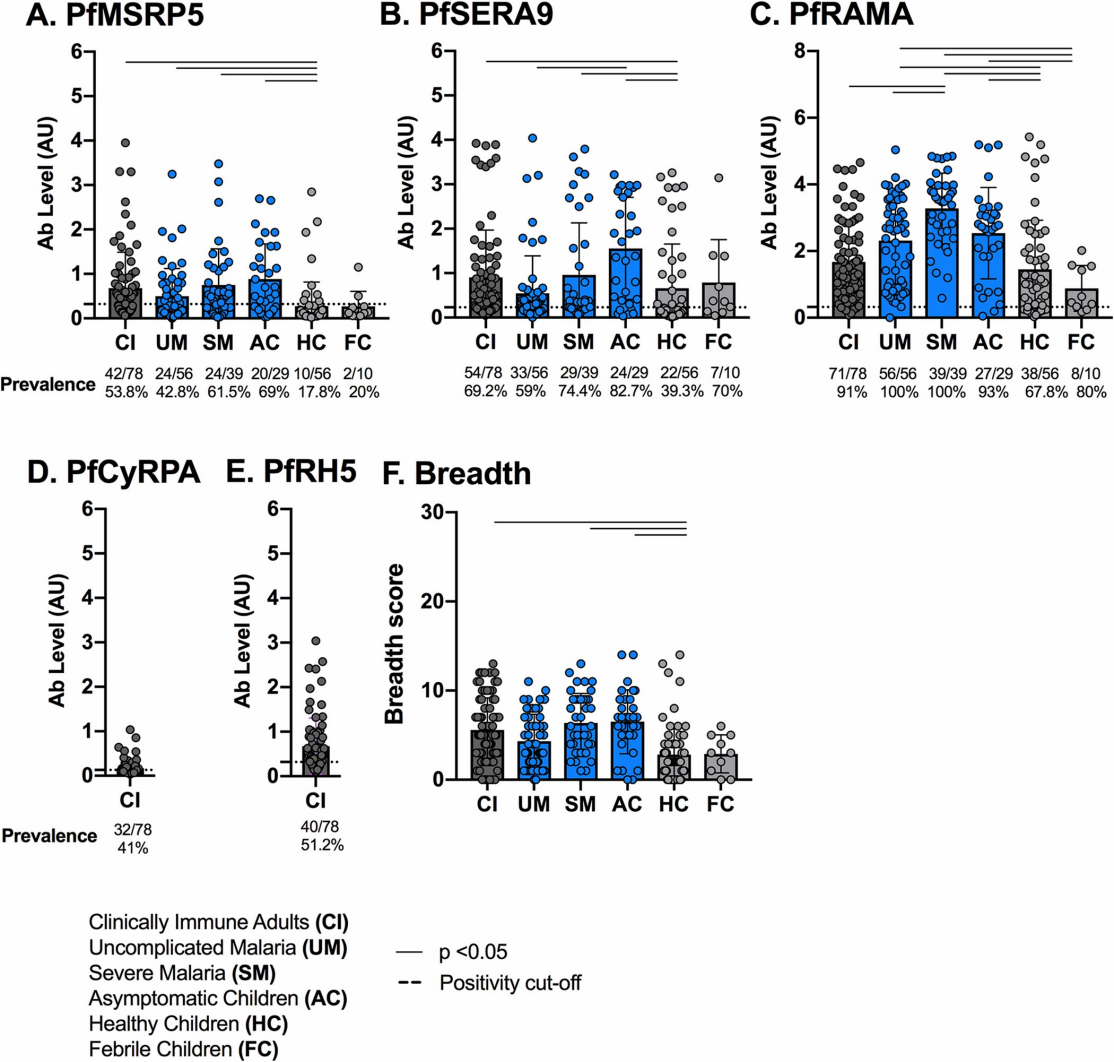
Prevalence and levels of IgG to *P*. *falciparum* merozoite antigens per clinical group. Levels (AU, mean with SD) of IgG specific for PfMSRP5 (A.), PfSERA9 (B.), PfRAMA (C.), PfCyRPA (D.) and PfRH5 (E.) were measured in clinically immune adults (CI, dark grey), children with *P*. *falciparum* malaria (asymptomatic children (AC), uncomplicated malaria (UM), severe malaria (SM), blue) and children without *P*. *falciparum* malaria (healthy children (HC), febrile children (FC), light grey). A Kruskal-Wallace test and Dunn’s post-hoc test were performed between all possible combinations of two clinical groups. Combinations with statistical significance only (p < 0.05) are represented by horizontal black lines. All p values regardless of statistical significance are shown in **S1 Table in S1 File.** Positivity cut-offs are represented by a dashed black line. For each clinical group, the number of positive individuals, total number of individuals tested and percent reactivity is shown at the bottom of each panel.

Next, we assessed PfMSRP5, PfSERA9 and PfRAMA-specific IgG levels in the six distinct groups **(Fig 3)**. IgG levels towards PfCyRPA and PfRH5 have been previously reported in all children used in this study [47], thus only PfCyRPA and PfRH5-specific IgG in CI adults was measured here **(Fig 3D and 3E)**. Overall, IgG prevalence and levels in uninfected healthy children (HC) were lower than those in clinically immune (CI) adults and in children with *P*. *falciparum* malaria (UM, SM and AC) **(Fig 3, S1 Table in S1 File)**. Specifically, for PfSERA9, levels of IgG were significantly higher in asymptomatic (AC) children than in children symptomatic uncomplicated malaria (UM) children **(Fig 3B, S1 Table in S1 File)**.

Breadth analysis of IgG showed that clinically immune adults (CI) and children with severe malaria (SM) and asymptomatic malaria (AC) had a broader response than healthy children (HC) **(Fig 3F, S1 Table in S1 File)**. These results suggest that children with *P*. *falciparum* malaria have higher merozoite antigen- specific IgG than children without *P*. *falciparum* malaria regardless of symptoms. Overall, the IgM and IgG responses to each of the antigens tested, mostly indicate parasite exposure.

### High levels of IgM are induced against multiple antigens in non-immune individuals

To better understand the contribution of IgG and IgM to protection, IgG and IgM responses within clinical categories for each antigen were compared. However, because of possible sensitivity differences of secondary detection antibodies and different affinities of the primary antibodies, caution must be taken when comparing IgM to IgG. To allow for direct comparisons the data was standardized and compared as level of antibody over background cut-off. IgG responses specific for PfCyRPA and PfRH5 in cohorts of children previously published [47] were included in this section.

**Fig 4:**
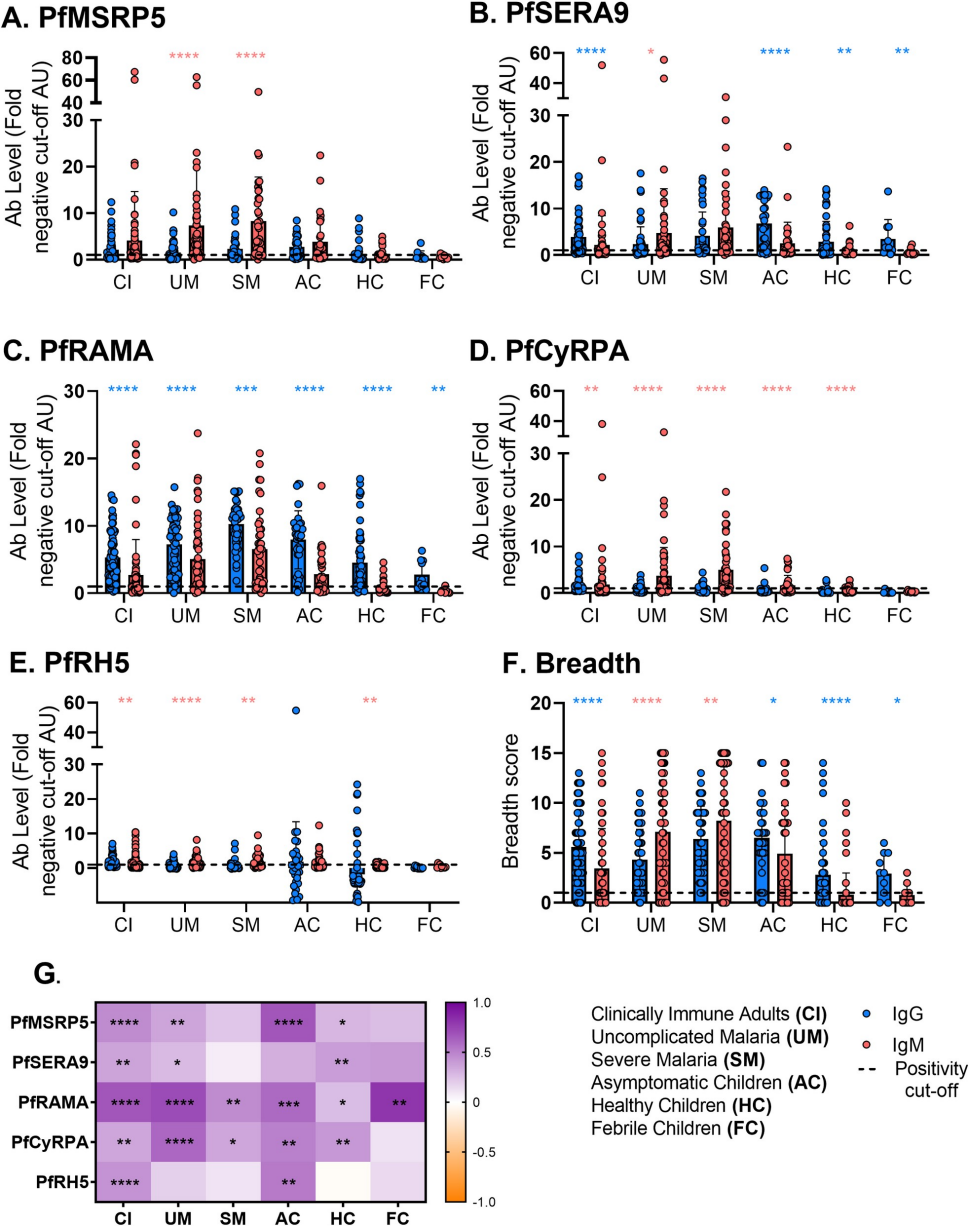
Comparison of levels and correlation of IgG and IgM to *P*. *falciparum* merozoite antigens per clinical group. Levels (fold over cut-off AU, mean with SD) of IgG (blue) and IgM (red) specific for PfMSRP5 (A.), PfSERA9 (B.), PfRAMA (C.), PfCyRPA (D.) and PfRH5 (E.) were measured in clinically immune adults (CI), asymptomatic children (AC), healthy children (HC), febrile children (FC) and children with uncomplicated (UM) and severe malaria (SM). Statistical significance (Wilcoxon test) between IgG and IgM is represented by asterisks (p<0.05 (*), p<0.01 (**), p<0.001 (***), p<0.0001 (****)). Asterisk colors represent whether IgG (blue) or IgM (red) is significantly higher. Exact p values are shown in **S2 Table in S1 File** (levels) and **S3 Table in S1 File** (prevalence). Spearman’s correlation of IgM and IgG responses for each antigen and clinical category is shown in the heat map (G). Statistical significance is represented by asterisks and color represents the strength and direction of correlation as shown in the key.

PfRAMA-specific IgG prevalence and levels were significantly higher than PfRAMA-specific IgM for all groups, indicating that regardless of clinical category PfRAMA induces a strong IgG responses **(Fig 4C, S2 and S3 Tables in S1 File**). For the remaining antigens, IgM prevalence and levels were higher than IgG prevalence and levels in children with acute *P*. *falciparum* malaria (SM and UM) **(Fig 4**). For CI adults, IgM prevalence and levels were higher than IgG prevalence and levels for PfCyRPA and PfRH5 **(Fig 4D and 4E**).

Breadth analysis further revealed that IgG responses were broader than IgM responses for subjects who were either aparasitemic or asymptomatic (CI, AC, HC, and FC) **(Fig 4F, S2 Table in S1 File**). For subjects with symptomatic *P*. *falciparum* malaria (SM and UM), IgM responses were significantly broader than IgG responses **(Fig 4, S2 Table in S1 File**) indicating that high levels of IgM are induced against multiple antigens in non-immune individuals.

To investigate the potential importance of IgM alongside IgG, IgM and IgG responses were assessed for correlation between antigens **(Fig 4G)**. Correlation was observed for all antigens for clinically immune adults (CI), children with UM (with the exception of PfRH5) and children with asymptomatic malaria (AC) (with the exception of PfSERA9). For subjects with severe malaria (SM), correlation was observed for PfRAMA and PfCyRPA only. These results suggest co-occurrence of IgM and IgG in subjects with less severe disease. The co-occurrence of IgM and IgG in subjects with less severe disease observed here is consistent with the limited published data on IgM [25].

### High parasitemia is associated with high levels of IgM

Higher merozoite antigen-specific IgG and IgM responses have been shown to be associated with both age and lower parasitemia [25, 33]. To better understand acquisition of antibody towards PfMSRP5, PfSERA9, PfRAMA, PfCyRPA and PfRH5, we assessed IgG and IgM levels relative to both age and parasitemia in children with acute *P*. *falciparum* malaria (SM and UM group).

**Fig 5:**
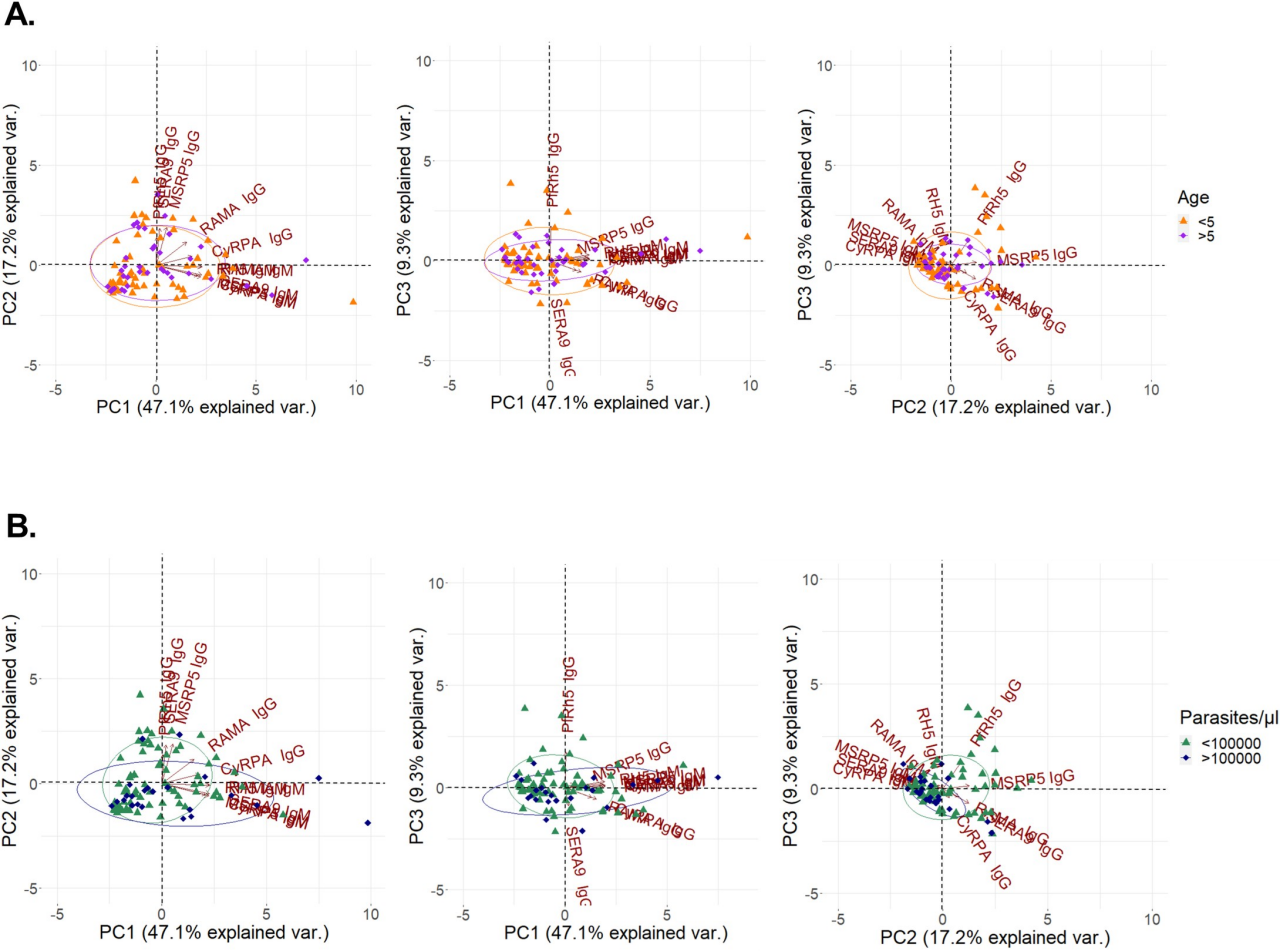
Principal components analysis (PCA) of antibody responses in subjects with acute *P*. *falciparum* malaria. Panels A and B show the plots of the distribution of individuals (SM and UM subjects). The first three principle components (PC1, PC2 and PC3), explaining the greatest variance, were chosen for representation, and include all antigen and antibody combinations tested. Panel A is grouped by age (<5; orange, >5; purple) whereas panel B is grouped by parasitemia (<100000 parasites/μL; green, >100000 parasites/μL; navy).

First, the relationship between age and IgM and IgG responses were examined. IgM levels did not correlate with age for any of the merozoite antigens tested (**S4 Table in S1 File**). Additionally, levels of IgG specific for PfMSRP5, PfSERA9 and PfRAMA did not increase significantly with age (**S4 Table in S1 File**). To assess age and antibody responses globally, a PCA analysis was performed. This analysis included previously published IgG responses to PfCyRPA and PfRH5 [47]. Results showed that antibody responses did not cluster differently when partitioned into subjects that were less than 5 years old and greater than 5 years old (**Fig 5A**).

**Fig 6:**
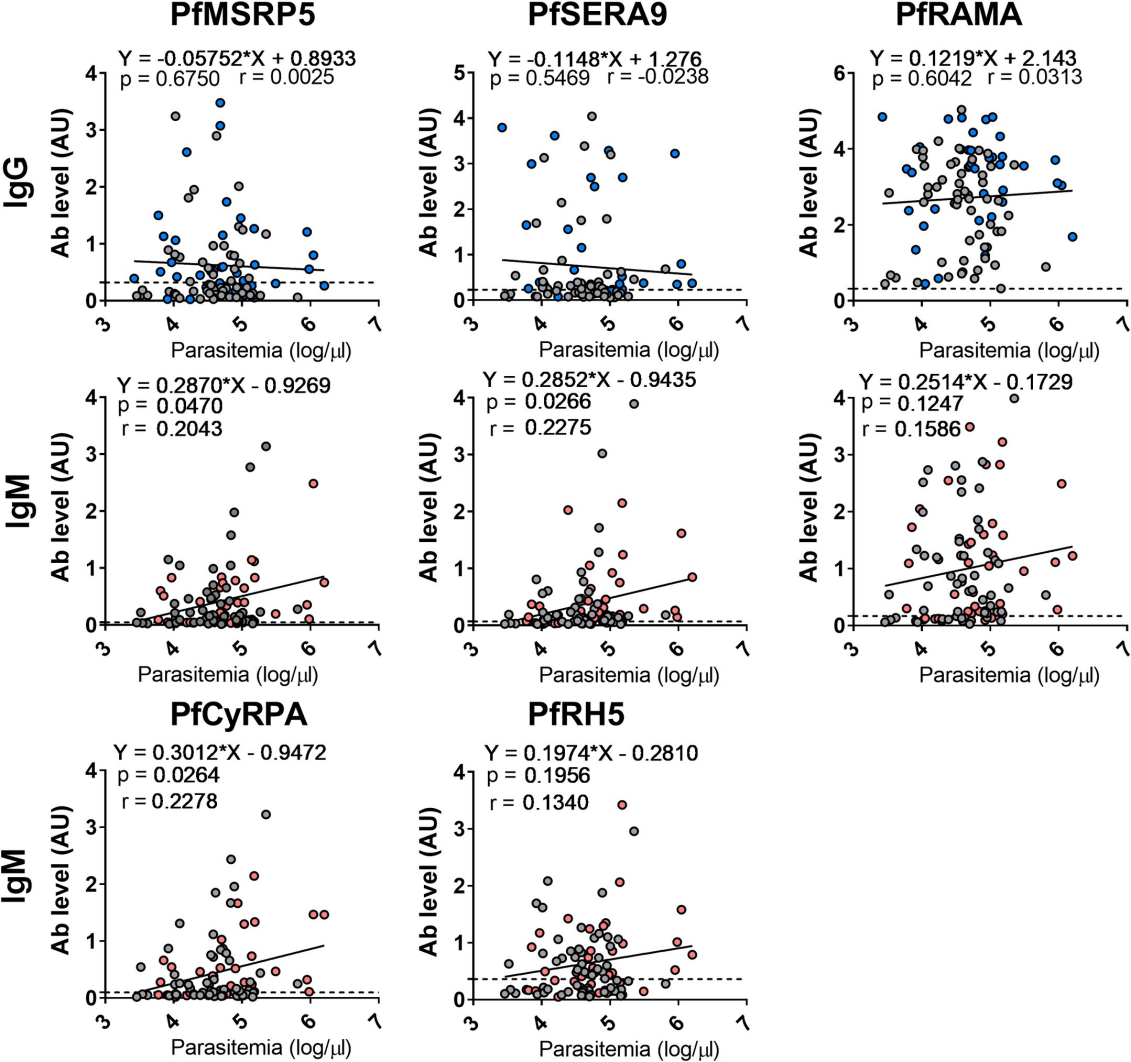
Relationship between parasitemia (log/μL) and isotype responses towards specific merozoite antigens. The relationship between parasitemia (log/μL, Y axis) and isotype level (AU) specific for PfMSRP5, PfSERA9, PfRAMA, PfCyRPA and PfRH5, were measured by linear regression (black line) in children with acute *P*. *falciparum* malaria (SM; IgG blue, IgM red circles and UM; grey circles). Negative cut-offs for each antigen are represented by a dashed line.

Next, the relationship between parasitemia and Ig-specific merozoite antigen responses were examined **(Fig 6)**. When IgM was assessed, an increase in parasitemia weakly correlated with higher IgM levels for PfMSRP5, PfSERA9 and PfCyRPA. Although no significant correlation was observed for PfRAMA and PfRH5, a similar trend was observed where IgM levels increased as parasitemia increased. We next assessed association for SM and UM groups separately and found that only IgM responses towards PfSERA9 and PfCyRPA differed between groups. For both PfSERA9 and PfCyRPA, SM groups were weakly significantly correlated whereas UM groups were not significantly correlated **(S1 Fig in S1 File)**. No significant correlation was observed for IgG towards any of the groups (UM, SM) or antigens tested (PfMSRP5, PfSERA9, PfRAMA) and parasitemia.

To assess the relationship between parasitemia and antibody responses to the merozoite antigens studied here, a PCA analysis was performed. This analysis included previously published IgG responses to PfCyRPA and PfRH5 [47]. Analysis by PCA **(Fig 5B)** showed that subjects with lower parasitemia (<100000 parasites/ul) clustered together and were partially segregated by IgG specific for PfMSRP5, PfSERA9, PfRAMA and PfRH5, although there was some overlap. This suggests that IgG reactivity to multiple antigens is associated with lower parasitemia.

To determine whether certain combinations of antigen-specific IgG or IgM responses affected parasitemia, we compared parasitemia_(log10) between negative and positive responders for all antigen combinations possible. This was performed on subjects with acute *P*. *falciparum* malaria using Wilcoxon test and Benjamini–Hochberg adjusted P values (false discovery rates). Although IgM responses to MSRP5, RAMA, CyRPA, MSRP5+RAMA, MSRP5+CyRPA, SERA9+CyRPA, RAMA+CyRPA, MSRP5+RAMA+CyRPA and SERA9+RAMA+CyRPA (**S5 Table in S1 File**) were associated with higher parasitemia, and IgG responses to MSRP5+SERA9+RH5 and MSRP5+SERA9+RAMA+RH5 (**S6 Table in S1 File**) were associated with lower parasitemia, these associations were not significant after adjusting for false discovery rate.

### Merozoite antigen-specific IgM induction and decline occurs earlier in infection than that of IgG

**Fig 7:**
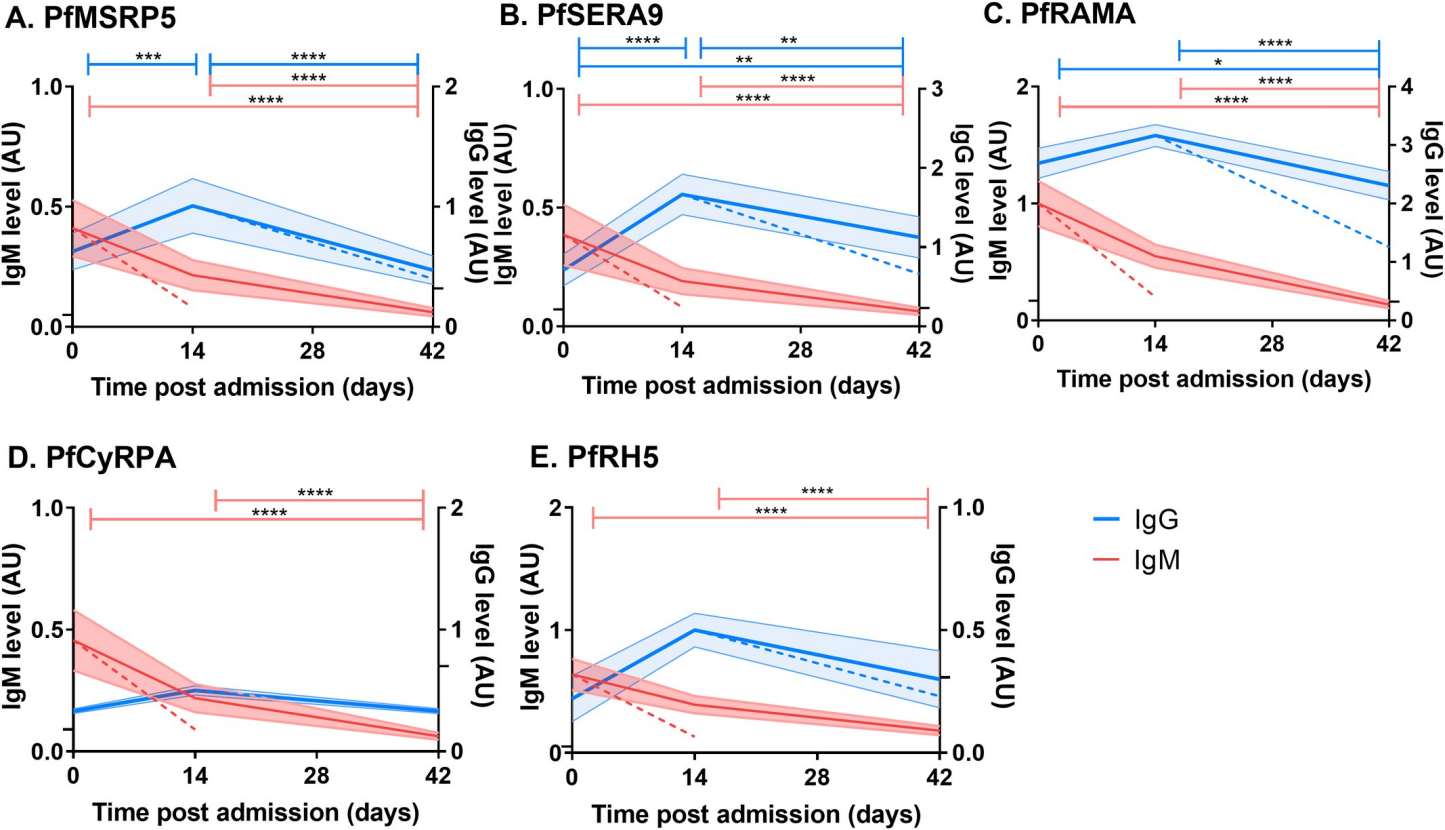
Longitudinal IgM and IgG specific for merozoite antigens in children with acute *P*. *falciparum* malaria. IgG (blue) and IgM (red) specific for PfMSRP5 (A.), PfSERA9 (B.) PfRAMA (C.), PfCyRPA (D.) and PfRH5 (E.) were measured longitudinally in children with acute *P*. *falciparum* malaria at day of diagnosis (Day 0), 2 weeks post-diagnosis (Day 14) and 6 weeks post-diagnosis (Day 42). Previously published IgG data for PfCyRPA and PfRH5 is included in the figure for visual comparison [47]. Cohort running means (thick line), their 95% confidence interval (shading) and catabolic decay (colored dashed lines) are shown. Longitudinal statistical significance (Friedman and Dunn’s post-hoc test) is represented by both asterisks (p<0.05 (*), p<0.01 (**), p<0.001 (***), p<0.0001 (****)) and colored lines (blue for IgG and red for IgM). Negative cut-offs are represented by a tick on each Y axis.

We previously reported that IgG levels towards PfRH5 and PfCyRPA increased in a minority of children at 2 weeks post-diagnosis, but quickly declined, leading to the conclusion that IgG responses to these antigens were short-lived. To determine whether IgM responses specific for the merozoite antigens PfMSRP5, PfSERA9, PfRAMA, PfCyRPA, PfRH5, and IgG responses specific for the merozoite antigens PfMSRP5, PfSERA9, PfRAMA were sustained or transient, IgM and IgG levels to each antigen were measured longitudinally in children with acute *P*. *falciparum* malaria (SM and UM). Responses were measured at 2 weeks (Day 14) and 6 weeks (Day 42) after initial diagnosis and sampling (Day 0) (**Fig 7**).

IgM declined significantly throughout follow-up with responses to all antigens significantly lower on Day 42 compared to those on Day 0 and Day 14 (**Fig 7)**. Additionally, when slopes were compared, responses to PfMSRP5 (p = 0.1732), PfSERA9 (p = 0.2940) and PfCyRPA (p = 0.2141) followed that of the predicted half-life of IgM whereas responses to PfRAMA (p = 0.0330) and PfRH5 (p = 0.0151) declined more slowly than predicted half-life. Nevertheless, by Day 42, mean responses for PfRH5 and PfRAMA were below cut-offs.

IgG levels increased significantly from diagnosis (Day 0) to 14 days post-diagnosis for PfMSRP5 and PfSERA9 (**Fig 7**). An increase was also observed for PfRAMA, however, this finding was not significant. The increase of IgG levels at Day 14 was followed by a significant decline in IgG levels to all antigens at 42 days post-diagnosis (**Fig 7**). When slopes were compared, responses to PfMSRP5 were not significantly different to that of predicted IgG half-life (p = 0.5887), whereas IgG responses towards PfSERA9 and PfRAMA declined more slowly than predicted by the IgG metabolic half-life (PfSERA9; p = 0.0123, PfRAMA; p = <0.0001). Furthermore, PfSERA9 responses at Day 42 were significantly higher than those at time of diagnoses (**Fig 7**). These results show that in children IgG responses to PfMSRP5 are transient, whereas IgG responses specific for PfSERA9 and PfRAMA do not rapidly decline following acute *P*. *falciparum* malaria. This suggests that long-lived plasma cells are induced only in response to the latter antigens. Overall, we observed that IgM responses in children to the antigens tested here were transient, supporting the general view that IgM induction and decline occur earlier in infection than that of IgG [53–55].

## Discussion

This study is the first to examine both IgG and IgM responses towards the merozoite antigens PfMSRP5, PfSERA9, PfRAMA, PfCyRPA and PfRH5. We found that IgG and IgM levels were highest in subjects with acute *P*. *falciparum* malaria with high IgM levels associated with higher parasitemia. However, following high IgM levels in children, IgM declined earlier in infection than that of IgG.

The majority of studies on malaria-induced humoral response have focused on understanding IgG dynamics and induction only, with little research investigating IgM responses. In this study, we found that children generally had higher IgM levels than clinically immune adults, with the exception of PfRH5-specific IgM. However, >25% of clinically immune adults had IgM to any of the antigens tested. This indicates that there may be some long-term IgM or IgM memory, and that IgM is a component of antibody responses to malaria even among those who are clinically immune.

Merozoite antigen-specific IgG prevalence observed in this study was similar to that observed in previous studies investigating the same antigens. PfRAMA-specific IgG has been found in approximately 85% of adults living in endemic areas and has been associated with protection and NAI to *P*. *falciparum* [33, 34, 42–44]. Prevalence studies on malaria exposed adults have found PfRH5-specifc IgG ranging from 30 – 91% of individuals tested [41, 56, 57]. Moreover, immune reactivity against both PfSERA9 and PfMSRP5 has been reported when examining subjects individually [33] and using pools of sera from exposed individuals [45]. For PfSERA9, this study supports previously published data that showed higher PfSERA9-specific IgG reactivity in asymptomatic children when compared to symptomatic children. This suggests some protective capacity of these antibodies [58]. In the current study we found that 41% of clinically immune adults had PfCyRPA-specific IgG, which was slightly higher than prevalence observed in children with severe malaria (28%) [47]. However, poor natural immunogenicity and a limited association with protection from disease have been described for PfCyPRA [33, 34, 59] thus, additional extensive studies should be performed to understand the role of this antigen in NAI.

In areas of intense *P*. *falciparum* transmission, antibodies to merozoite antigens are associated with lower parasitemia and clinical immunity in cohorts of older children [25, 33]. Recent findings using subjects from Papua New Guinea (PNG) have shown that levels of IgM against whole merozoites, and IgG towards PfRAMA and PfRH5, increase with age [25, 34]. Furthermore, IgG towards PfMSRP5, PfSERA9 and PfRAMA has also been reported to increase with age in uninfected highly exposed Malian individuals aged 3 months – 25 years old [33]. In the current study, we found no association between merozoite antigen-specific IgM responses, IgG responses and age to any of the antigens tested. These discrepant results could be due to differences in the age of the individuals in the study cohorts, endemicity and antigens tested.

In a cohort of children from PNG, IgG specific for PfRAMA and PfRH5 has been shown to be associated with protection [34]. However, consistent with our previous findings on IgG-specific for PfCyRPA and PfRH5 [47], parasitemia did not correlate with levels of IgG-specific for PfMSRP5, PfSERA9 and PfRAMA. Conversely, higher IgM was weakly correlated with higher parasitemia for PfMSRP5, PfSERA9 and PfCyRPA, primarily due to SM subjects, indicating that increased IgM levels are a marker of high parasite load.

It is generally believed that in *P*. *falciparum* malaria IgM responses are short-lived and that IgG responses are associated with protection and acquisition of NAI. However, a recent study in a murine malaria model demonstrated that IgM memory B cells were early rapid responders that initiated secondary responses [26]. Furthermore, these IgM responses were long-lived and therefore, it has been suggested that IgM may play an essential role in protection from malaria by maintaining and boosting IgG responses [25, 26]. In the current study IgM was detected in adults which may suggest some long term IgM or eventual acquisition of memory. Nevertheless, the longitudinal data in children presented here support the model observed in many viral infections where IgM is induced in early infection but is rapidly replaced by IgG [60]. This is in contrast to previous data demonstrating that IgM responses against merozoite antigen MSP2 were sustained in returned Australian travelers, and in children living in Kenya during a period of minimal malarial transmission [25]. This suggests that antibodies to the antigens tested here do not reflect the immune responses to all blood stage *P*. *falciparum* antigens [61, 62]. These differences in IgM maintenance and decay could be due to the specific merozoite antigens tested, endemicity and the intensity of *P*. *falciparum* transmission [61, 62]. MSP2 is a highly abundant protein and maintenance of IgM titers may require a higher (cumulative) amount of antigen [63]. Alternatively, the ability to boost memory IgM responses may be antigen dependent, reflecting structural differences and conservedness among parasite proteins. Additionally, the subjects tested in this study were located in Ghana in areas of intense *P*. *falciparum* transmission. This is considerably different from the cohorts of returned Australian travelers and Kenyan children used in the previously cited study where *P*. *falciparum* transmission was less intense [25]. Furthermore, IgG half-lives are thought to be shorter if measured after acute infection than measured in un-infected subjects [64–66], thus the acutely infected subject used here could also impact the findings presented on IgG maintenance and decay.

The results here suggest that malaria induced antibodies to PfMSRP5, PfSERA9, PfRAMA, PfCyRPA and PfRH5 may be secreted by short-lived plasma cells or by B1 cells [67–69], rather than by conventional memory B cells. Short-lived plasma cells and B1 cells can respond to a second antigen encounter in a way that is similar to the primary response, undergoing isotype switching from IgM to IgG. This is evidenced by clinically immune adults presenting both IgM and IgG, and by high IgM and IgG levels occurring in subjects with acute *P*. *falciparum* malaria, where IgG is followed by IgM. Nevertheless, the IgM and IgG responses observed in clinically immune adults may also indicate that there is long term IgM or eventual acquisition of IgM memory. However, additional extensive studies will need to be performed to determine the role that IgM plays in NAI.

Although the results reported here are informative for *P*. *falciparum* malaria pathogenesis and vaccine research there are limitations. A key limitation to this study is a lack of clinical history for FC and HC groups that, although unlikely, may not have been exposed to *P*. *falciparum*. Furthermore, at the time of collection (Day 0) antibodies were already induced and so it would be beneficial to include a pre-infection time-point to correctly evaluate induction and waning. Additional longitudinal sampling would also help to strengthen this data as re-infection and further protection of antibody responses could be evaluated. Finally, as mentioned above, caution must be taken when comparing IgM and IgG responses due to possible differences in the sensitivity of detection antibodies.

Overall, after *P*. *falciparum* malaria exposure we observed high levels of IgM and IgG where IgM induction and decline occur earlier in infection than that of IgG. Although both IgM and IgG responses in children reported here are transient, vaccine induced antibodies against the five antigens analyzed, especially PfRH5, may prove more effective and reach protective levels [41]. However, further investigation on this topic is warranted. Additionally, it has been previously observed that the five parasite antigens used in this study give rise to growth inhibitory and synergistic antibodies *in vitro* [33]. This study highlights the need to characterize protective epitopes present in these antigens and investigate synergistic combinations of antibodies targeting these proteins, which may inform the design of a second-generation multivalent malaria vaccine. Furthermore, this study provides important insight into the acquisition and maintenance of IgM and IgG in adults and children in distinct clinical categories.

## Supporting information

S1 File (PDF)
