# Supplementary material for: Acquisition and decay of IgM and IgG responses to merozoite antigens after Plasmodium falciparum malaria in Ghanaian children
Source: PLoS One. 2020 Dec 17;15(12):e0243943. doi: 10.1371/journal.pone.0243943 (PMC7746192; doi:10.1371/journal.pone.0243943)
Supplement: S1 File — (PDF) [file pone.0243943.s001.pdf]

Supplementary information

S1 Table. Statistical significance of prevalence and levels of IgM and IgG specific for *P. falciparum* merozoite antigens between clinical groups

Group	MSRP5				SERA9				RAMA			
	IgG		IgM		IgG		IgM		IgG		IgM	
	P (Dunn's)	P (Fisher)	P (Dunn's)	P (Fisher)	P (Dunn's)	P (Fisher)	P (Dunn's)	P (Fisher)	P (Dunn's)	P (Fisher)	P (Dunn's)	P (Fisher)
**CI/AC**	>0.9999	0.1901	>0.9999	0.1156	0.8373	**0.0071**	>0.9999	0.1834	0.1131	**0.0114**	>0.9999	**0.0173**
**CI/HC**	**<0.0001**	**<0.0001**	**<0.0001**	**<0.0001**	**0.0021**	0.1157	0.0917	**0.0004**	>0.9999	**0.0013**	**<0.0001**	**0.0002**
**CI/FC**	0.5798	0.0892	0.2194	**0.0403**	>0.9999	0.5019	0.8983	0.4838	0.9655	0.2704	0.0978	**0.0482**
**CI/UM**	>0.9999	0.2249	**0.0383**	**0.0086**	0.0847	0.5997	**0.0016**	**<0.0001**	0.1342	0.1378	**0.0015**	**0.0004**
**CI/SM**	>0.9999	0.5533	**0.0020**	**0.0015**	>0.9999	**0.0443**	**<0.0001**	**<0.0001**	**<0.0001**	0.2628	**<0.0001**	**<0.0001**
**AC/HC**	**<0.0001**	**<0.0001**	**<0.0001**	**<0.0001**	**<0.0001**	**0.0002**	**0.0085**	**<0.0001**	**0.0028**	>0.9999	**<0.0001**	**<0.0001**
**AC/FC**	0.1725	**0.0107**	**0.0422**	**0.003**	>0.9999	0.3989	0.1726	0.0569	**0.0163**	0.6927	**0.0067**	**0.0022**
**AC/UM**	0.2089	**0.0385**	>0.9999	0.7801	**0.0009**	**0.0305**	>0.9999	0.0596	>0.9999	**0.0002**	>0.9999	0.8002
**AC/SM**	>0.9999	0.6122	0.6195	0.3357	>0.9999	0.5567	0.0899	0.0735	0.3668	**0.0012**	0.3766	0.2543
**HC/FC**	>0.9999	>0.9999	>0.9999	0.6685	>0.9999	0.0916	>0.9999	0.2853	>0.9999	0.7108	>0.9999	>0.9999
**HC/UM**	**0.0097**	**0.0071**	**<0.0001**	**<0.0001**	>0.9999	0.0583	**<0.0001**	**<0.0001**	**0.0019**	**<0.0001**	**<0.0001**	**<0.0001**
**HC/SM**	**<0.0001**	**<0.0001**	**<0.0001**	**<0.0001**	**0.0289**	**0.0009**	**<0.0001**	**<0.0001**	**<0.0001**	**0.0002**	**<0.0001**	**<0.0001**
**FC/UM**	>0.9999	0.2931	**0.0013**	**0.0004**	>0.9999	0.728	**0.0020**	**0.0023**	**0.0244**	0.056	**<0.0001**	**0.0003**
**FC/SM**	0.3437	**0.0315**	**0.0001**	**0.0001**	>0.9999	>0.9999	**0.0001**	**0.0027**	**<0.0001**	0.098	**<0.0001**	**<0.0001**
**UM/SM**	0.5489	0.0959	>0.9999	0.42	0.3879	0.1202	>0.9999	0.9008	**0.0351**	>0.9999	>0.9999	0.3152

S1 Table. continued

Group	CyRPA		RH5		Breadth	
	IgM		IgM		IgG	IgM
	P (Dunn's)	P (Fisher)	P (Dunn's)	P (Fisher)	P (Dunn's)	P (Dunn's)
**CI/AC**	>0.9999	0.0975	>0.9999	0.5028	>0.9999	>0.9999
**CI/HC**	**0.0002**	**0.0236**	**<0.0001**	**0.0026**	**<0.0001**	**0.0001**
**CI/FC**	0.5412	0.4441	0.4035	0.4838	0.4671	>0.9999
**CI/UM**	**0.0032**	**<0.0001**	0.5987	**0.0341**	0.9706	**0.0033**
**CI/SM**	**<0.0001**	**<0.0001**	0.1891	**0.0289**	>0.9999	**0.0003**
**AC/HC**	**0.0006**	**0.001**	**<0.0001**	**0.0023**	**<0.0001**	**<0.0001**
**AC/FC**	0.2761	0.0678	0.0905	0.2625	0.1029	0.3061
**AC/UM**	0.3586	0.1695	>0.9999	0.3716	0.134	>0.9999
**AC/SM**	**0.0249**	**0.0274**	>0.9999	0.3271	>0.9999	0.2597
**HC/FC**	>0.9999	>0.9999	>0.9999	0.6162	>0.9999	>0.9999
**HC/UM**	**<0.0001**	**<0.0001**	**<0.0001**	**<0.0001**	0.0773	**<0.0001**
**HC/SM**	**<0.0001**	**<0.0001**	**<0.0001**	**<0.0001**	**<0.0001**	**<0.0001**
**FC/UM**	**0.0012**	**0.0028**	**0.0196**	0.0824	>0.9999	**0.0057**
**FC/SM**	**<0.0001**	**0.0005**	**0.0076**	**0.0374**	0.095	**0.0011**
**UM/SM**	>0.9999	0.3058	>0.9999	0.7262	0.0951	>0.9999

S2 Table. Statistical significance between IgG and IgM levels specific for *P. falciparum* merozoite antigens

Group	MSRP5	SERA9	RAMA	CyRPA	RH5	Breadth
**CI**	0.3177	**<0.0001**	**<0.0001**	**0.0095**	**0.0033**	**<0.0001**
**UM**	**<0.0001**	**0.0211**	**<0.0001**	**<0.0001**	**0.0001**	**<0.0001**
**SM**	**<0.0001**	0.1711	**0.0002**	**<0.0001**	**0.0024**	**0.0026**
**AC**	0.1386	**<0.0001**	**<0.0001**	**<0.0001**	0.126	**0.028**
**HC**	0.9002	**0.003**	**<0.0001**	**<0.0001**	**0.004**	**<0.0001**
**FC**	0.1602	**0.0059**	**0.002**	0.084	0.084	**0.0117**

S3 Table. Comparison of IgG and IgM prevalence specific for *P. falciparum* merozoite antigens

		CI	UM	SM	AC	HC	FC
**No. total**		**78**	**56**	**39**	**29**	**56**	**10**
**MSRP5**	**IgM (%)**	45 (57.7)	45 (80.4)	34 (87.2)	22 (75.8)	9 (16)	2 (20)
	**IgG (%)**	42 (53.8)	24 (42.8)	24 (61.5)	20 (69)	10 (17.8)	2 (20)
	**p value**	0.7473	**<0.0001**	**0.0183**	0.07696	>0.9999	>0.9999
**SERA9**	**IgM (%)**	28 (35.9)	41 (73.2)	29 (74.4)	15 (51.7)	5 (8.9)	2 (20)
	**IgG (%)**	54 (69.2)	33 (59)	29 (74.4)	24 (82.7)	22 (39.3)	7 (70)
	**p value**	**<0.0001**	0.162	>0.9999	**0.0239**	**0.0003**	0.0698
**RAMA**	**IgM (%)**	33 (42.3)	41 (73.2)	32 (82)	20 (68.9)	7 (12.5)	1 (10)
	**IgG (%)**	71 (91)	56 (100)	39 (100)	27 (93)	38 (67.8)	8 (80)
	**p value**	**<0.0001**	**<0.0001**	**0.0116**	**0.0411**	**<0.0001**	**0.0055**
**CyRPA**	**IgM (%)**	19 (24.4)	33 (58.9)	27 (69.2)	12 (41.3)	5 (8.9)	0 (0)
	**IgG (%)**	32 (41)	– [a]	– [a]	– [a]	– [a]	– [a]
	**p value**	**0.04**	**<0.0001**	**0.0006**	**0.0148**	0.4379	>0.9999
**RH5**	**IgM (%)**	28 (35.9)	31 (55.4)	23 (58.9)	13 (44.8)	7 (12.5)	2 (20)
	**IgG (%)**	40 (51.2)	– [a]	– [a]	– [a]	– [a]	– [a]
	**p value**	0.0754	**0.0004**	**0.0025**	0.7901	0.4410	0.4737

[a] See Partey et al [47]

S4 Table. Correlation of antibody levels to age using Spearman's correlation co-efficient

	IgM		IgG	
	**p**	**r**	**p**	**r**
**PfMSRP5**	0.3124	0.1065	0.0807	0.0183
**PfSERA9**	0.9839	0.0021	0.04568	0.0785
**PfRAMA**	0.561	0.0614	0.4778	0.0749
**PfCyRPA**	0.9425	0.0076	NA [a]	NA
**PfRH5**	0.0531	0.2023	NA	NA

[a] Not available. See Partey et al [47]

S5 Table. Comparison of parasitemia in individuals with positive or negative IgM responses to *P. falciparum* antigens alone or in combination

	Negative			Positive			P value	BH[b]- adjusted
	Parasitemia (log10)	SD[a]	sample size	Parasitemia (log10)	SD	sample size		
**MSRP5**	4.35	0.54	16	4.73	0.54	76	**0.0294**	0.1280
SERA9	4.49	0.54	24	4.73	0.55	68	0.1927	0.2783
**RAMA**	4.35	0.50	20	4.75	0.54	72	**0.0065**	0.0975
**CyRPA**	4.47	0.49	33	4.78	0.56	59	**0.0226**	0.1280
PfRh5	4.57	0.56	41	4.75	0.54	51	0.2783	0.2783
MSRP5 + SERA9	4.49	0.50	27	4.74	0.56	65	0.1186	0.2541
**MSRP5 + RAMA**	4.36	0.50	22	4.76	0.54	70	**0.0050**	0.0975
SERA9 + RAMA	4.49	0.46	26	4.74	0.56	66	0.1077	0.2485
**MSRP5 + CyRPA**	4.48	0.48	34	4.78	0.56	58	**0.0299**	0.1280
**SERA9 + CyRPA**	4.49	0.50	35	4.78	0.56	57	**0.0427**	0.1423
**RAMA + CyRPA**	4.47	0.48	34	4.78	0.56	58	**0.0196**	0.1280
SERA9 + PfRh5	4.57	0.56	41	4.75	0.54	51	0.2783	0.2783
MSRP5 + PfRh5	4.57	0.54	42	4.75	0.55	50	0.2259	0.2783
RAMA + PfRh5	4.56	0.52	43	4.76	0.55	49	0.1668	0.2780
CyRPA + PfRh5	4.59	0.48	49	4.76	0.58	43	0.2747	0.2783
MSRP5 + SERA9 + RAMA	4.48	0.48	28	4.75	0.56	64	0.0827	0.2067
MSRP5 + SERA9 + CyRPA	4.50	0.49	36	4.77	0.57	56	0.0544	0.1482
**MSRP5 + RAMA + CyRPA**	4.48	0.48	35	4.78	0.57	57	**0.0259**	0.1280
**SERA9 + RAMA + CyRPA**	4.49	0.47	35	4.78	0.56	57	**0.0427**	0.1423
MSRP5 + SERA9 + PfRh5	4.57	0.54	42	4.75	0.55	50	0.2259	0.2783
MSRP5 + RAMA + PfRh5	4.56	0.53	43	4.76	0.55	49	0.1668	0.2780
SERA9 + RAMA + PfRh5	4.56	0.51	43	4.76	0.55	49	0.1668	0.2780
MSRP5 + CyRPA + PfRh5	4.59	0.49	49	4.76	0.58	43	0.2747	0.2783
SERA9 + CyRPA + PfRh5	4.59	0.47	49	4.76	0.58	43	0.2747	0.2783
RAMA + CyRPA + PfRh5	4.59	0.49	49	4.76	0.58	43	0.2747	0.2783

	Negative			Positive			P value	BH[b]- adjusted
	Parasitemia (log10)	SD[a]	sample size	Parasitemia (log10)	SD	sample size		
MSRP5 + SERA9 + RAMA + CyRPA	4.50	0.50	36	4.77	0.57	56	0.0544	0.1482
MSRP5 + SERA9 + RAMA + PfRh5	4.59	0.48	49	4.76	0.58	43	0.1668	0.2780
MSRP5 + RAMA + CyRPA + PfRh5	4.59	0.48	49	4.76	0.58	43	0.2747	0.2783
SERA9 + RAMA + CyRPA + PfRh5	4.59	0.48	49	4.76	0.58	43	0.2747	0.2783
MSRP5 + SERA9 + RAMA + CyRPA + PfRh5	4.59	0.48	32	4.76	0.58	43	0.2747	0.2783

[a] SD, standard deviation; BH[b], benjamini–hochber

S6 Table. Comparison of parasitemia in individuals with positive or negative IgG responses to *P. falciparum* antigens alone or in combination

Combination	Negative			Positive			P value	BH[b]- adjusted
	Parasitemia (log10)	SD[a]	sample size	Parasitemia (log10)	SD	sample size		
MSRP5	4.67	0.56	48.00	4.67	0.55	44	0.7586	0.7586
SERA9	4.64	0.48	37.00	4.69	0.60	55	0.7501	0.7586
CyRPA	4.63	0.55	73.00	4.82	0.55	19	0.3445	0.42106
PfRh5	4.71	0.57	73.00	4.49	0.47	19	0.1864	0.38432
MSRP5 + SERA9	4.69	0.53	54.00	4.64	0.58	38	0.3433	0.42106
MSRP5 + RAMA	4.67	0.56	48.00	4.67	0.55	44	0.7586	0.7586
SERA9 + RAMA	4.64	0.48	37.00	4.69	0.60	55	0.7501	0.7586
MSRP5 + CyRPA	4.63	0.54	79.00	4.90	0.61	13	0.2261	0.38432
SERA9 + CyRPA	4.63	0.54	77.00	4.86	0.61	15	0.2671	0.41973
RAMA + CyRPA	4.63	0.55	73.00	4.82	0.55	19	0.3445	0.42106
SERA9 + PfRh5	4.72	0.55	77.00	4.41	0.47	15	0.0659	0.36245
MSRP5 + PfRh5	4.72	0.56	75.00	4.45	0.49	17	0.1075	0.38432
RAMA + PfRh5	4.71	0.57	73.00	4.49	0.47	19	0.1864	0.38432
MSRP5 + SERA9 + RAMA	4.69	0.53	54.00	4.64	0.58	38	0.3433	0.42106
MSRP5 + SERA9 + CyRPA	4.63	0.53	79.00	4.90	0.61	13	0.2261	0.38432
MSRP5 + RAMA + CyRPA	4.63	0.54	79.00	4.90	0.61	13	0.2261	0.38432
SERA9 + RAMA + CyRPA	4.63	0.54	77.00	4.86	0.61	15	0.2271	0.38432
**MSRP5 + SERA9 + PfRh5**	4.72	0.55	78.00	4.38	0.48	14	**0.04975**	0.36245
MSRP5 + RAMA + PfRh5	4.72	0.56	75.00	4.45	0.49	17	0.1075	0.38432
SERA9 + RAMA + PfRh5	4.72	0.55	77.00	4.41	0.47	15	0.0659	0.36245
MSRP5 + SERA9 + RAMA + CyRPA	4.63	0.53	79.00	4.90	0.61	13	0.2261	0.38432
**MSRP5 + SERA9 + RAMA + PfRh5**	4.72	0.55	78.00	4.38	0.48	14	**0.04975**	0.36245

[a] SD, standard deviation; BH[b], benjamini–hochberg

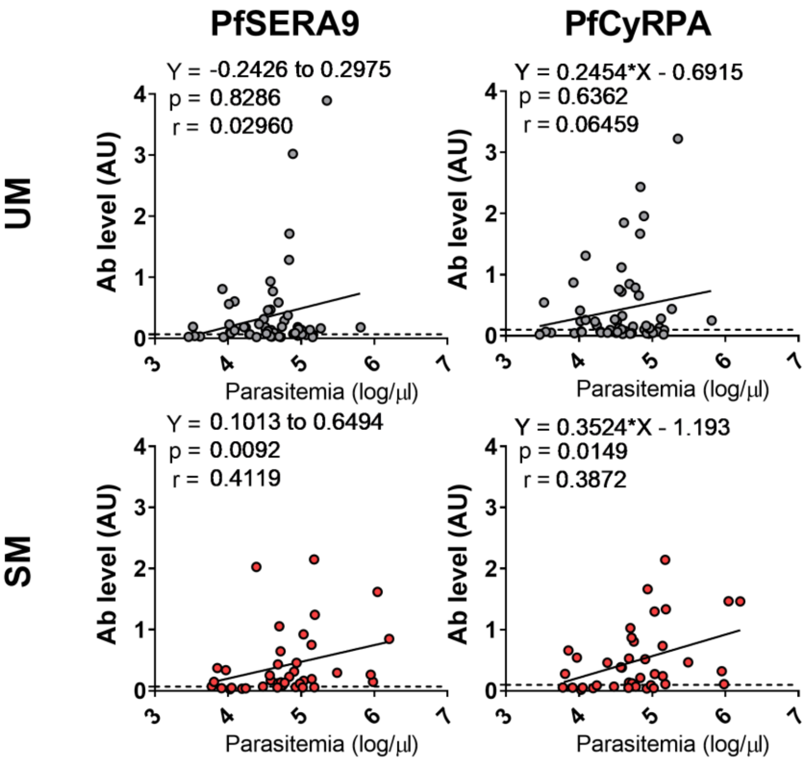

S1 Fig. Relationship between parasitemia (log/µL) and IgM responses towards specific merozoite antigens. The relationship between parasitemia (log/µL, Y axis) and IgM level (AU) specific for PfSERA9 and PfCyRPA, were measured by linear regression (black line) in children with acute *P. falciparum* malaria (SM; red circles and UM; grey circles). Negative cut-offs for each antigen are represented by a dashed line.
